# Negotiating grey areas: an interview-based analysis of paramedic uncertainty and decision-making in cardiac arrest events

**DOI:** 10.1186/s12873-024-01057-z

**Published:** 2024-08-29

**Authors:** Galina Gardiner, Karin Eli, Caroline J. Huxley, Rachael Fothergill, Gavin D. Perkins, Michael A. Smyth, Frances Griffiths, Anne-Marie Slowther

**Affiliations:** University of Warwick Medical School, Coventry, Warwickshire, UK

**Keywords:** Uncertainty, Decision making, Resuscitation, Cardiac arrest, Paramedic

## Abstract

**Background:**

Paramedics are responsible for critical resuscitation decisions when attending Out of Hospital Cardiac Arrests (OHCA). Existing research indicates that a range of clinical and non-clinical factors moderate their decision-making. Within the United Kingdom (UK), there is little evidence on how and why paramedics make their decisions at actual OHCA events.

**Methods:**

We explored the experiences of UK paramedics using individually recalled OHCA events as catalysts for discussion. Pen portraits developed from semi-structured interviews with 31 paramedics across two UK ambulance services were thematically analysed, enabling cross-participant comparisons whilst retaining depth and context.

**Results:**

We identified four themes: uncertainties encountered in resuscitation guidelines, influences on decision-making, holistic perspectives, and indirect moderators. We found that paramedics experienced uncertainty at all stages of the resuscitation process. Uncertainties arose from indeterminate, ambiguous or complex information and were described as having both clinical and ethical dimensions. Whilst guidelines drove paramedics’ decisions, non-clinical personal, practical and relational factors moderated their assessments of survivability and decision-making, with attitudes to interactions between patient age, frailty and quality of life playing a substantial role. Coping strategies such as uncertainty reduction, assumption-based reasoning and weighing pros and cons were evident from interviews.

**Conclusions:**

The complexity of interactions between clinical and non-clinical factors points to an element of variability in paramedics’ responses to uncertainty. Exploring UK paramedics’ uncertainties and decision-making during specific OHCA events can help acknowledge and address uncertainties in resuscitation guidelines and paramedic training, providing paramedics with the tools to manage uncertainty in a consistent and transparent way.

## Background

Cardiac arrest happens when the heart abruptly ceases pumping blood around the body. When out-of-hospital cardiac arrests (OHCA) occur, paramedics are called upon to make urgent life and death decisions. In England, paramedics are called to around 100,000 OHCA each year and perform resuscitation where there is any chance of survival. Resuscitation is attempted in around 35,000 patients, around 58% of whom are declared dead at the scene and 42% transported to hospital, with survival rates of approximately 7–9% [1–3].

Paramedics attending OHCA aim to optimise patients’ chances of recovery without causing unnecessary suffering if resuscitation would be unsuccessful. They face a range of uncertainties in determining survivability (the likelihood that a patient will survive the arrest) and when making decisions about whether to terminate a resuscitation attempt. The UK national Recognition of Life Extinct (ROLE) guideline [2] helps paramedics navigate these uncertainties, aiming to provide direction and consistency by informing decisions at all stages of an event: in starting resuscitation (or continuing if bystanders have begun), and in deciding whether to continue treatment on scene, transport, or cease resuscitation.

To enhance readers’ understanding of this study, key guideline criteria and relevant clinical information are described in Box 1.

**Box. 1:**
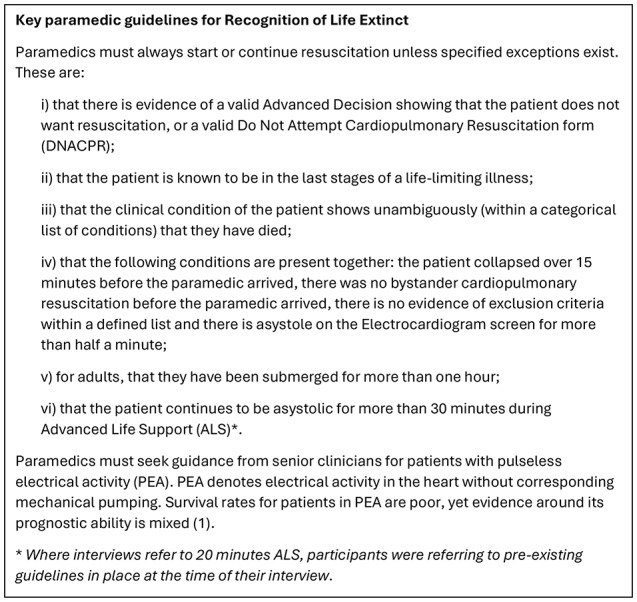
Guidelines and clinical information relevant to study (2)

Historically, most research on paramedic decision-making during OHCA has been conducted using data derived from cardiac arrest registries or clinical observations [4]. However, recent qualitative research focusing on paramedics’ perspectives in several countries has highlighted the importance of both clinical and non-clinical factors in influencing resuscitation decision-making [5–7]. Non-clinical factors were explored further by Milling et al. [8] in a mixed-methods systematic review which identified substantial roles played by external, ethical, emotional and relational aspects of the decision-making process. Within the UK, a small number of studies have explored paramedic decision-making. Two of these used hypothetical vignette-based scenarios [5, 9] and one involved interviews with senior clinicians about resuscitation decisions for patients in a PEA rhythm [10]. Brandling et al. [5] found that personal, interpersonal, cultural and risk factors affected UK paramedics’ decision-making at all stages of a hypothetical OHCA event. Although contentious, paramedics’ discussions suggested that culturally accepted norms of life expectancy influenced paramedic behaviour [5]. This factor was also considered within a holistic framework of decision-making for senior paramedics when deciding whether to cease resuscitation [10] and was associated with paramedic decisions to attempt resuscitation in discrete choice vignette scenarios [9]. Our study builds on this work by exploring UK paramedics’ individual experiences of uncertainty and decision-making at specific OHCA events.

We know from the literature [5–12] that resuscitation decision-making is complex, and that considerations other than those that can be algorithmically addressed often enter into the process. What we don’t know is how paramedics manage the multiple variables involved in this complex decision-making process, and how they reflect on and experience these processes.

This paper conceptualises paramedics’ experiences within the wider context of uncertainty and decision-making in healthcare. Often described as conscious awareness of lack of knowledge or ‘subjective perception of ignorance’ [13], uncertainty can inhibit clinicians’ confidence in diagnosis, treatment and prognosis at both a cognitive and existential level [14]. Although the relationships between uncertainty and decision-making are not fully understood, the identification of factors moderating healthcare workers’ responses to uncertainty is key to understanding how it is managed [15]. In emergency settings where time is pressured and stakes are high, the type and source of uncertainty faced by emergency workers is intrinsically linked to their decision-making process [16]. Naturalistic decision-making research, which uses real-world settings to explore complex decision-making, indicates that emergency workers’ coping strategies utilise the RAWFS heuristic [17]: Reduction of uncertainty, Assumption-based reasoning, Weighing pros/cons of competing alternatives, Forestalling/hedging, and Suppressing uncertainty. This framework has been used to investigate a range of scenario-based decision-making, from emergency services to air traffic control [18–20]. It has once been applied to paramedics attending non-routine situations [21], finding their coping strategies to be associated with both the type of uncertainty (inadequate information) being experienced and the phase of the event, with uncertainty reduction strategies used most frequently. To date, there has been no research focusing on UK paramedics’ perspectives of uncertainty and decision-making in specific OHCA events.

We carried out a study to explore the experiences and perspectives of UK paramedics responding to specific OHCA events, focusing on the decision whether or not to terminate resuscitation. This was a specific discrete project conducted under the auspices of a larger study which contained separate statistical and economic work packages examining termination of resuscitation rules [22]. In this paper we explore how paramedics experience uncertainty and the factors that moderate this uncertainty and impact their decision-making throughout an OHCA event. Our exploration of paramedics’ experiences can be used to inform revisions to UK guidelines and training for paramedics.

Two main questions are addressed:

1. How and when do paramedics describe encountering uncertainty when managing Out of Hospital Cardiac Arrests?
2. What are the factors that paramedics perceive to moderate these uncertainties, influencing their assessments of survivability and affecting their decisions?

## Methods

### Study design

The study was conducted from a critical realist perspective. The critical realist position acknowledges researcher and participant subjectivity, particularly relevant when addressing a sensitive and traumatic topic in which participants may feel their professional expertise and personal values to be under interrogation [23]. It sees subjective descriptions as valid windows on an external world, enabling findings to be analysed with a view to impacting policy and research [24]. With this in mind we used thematic analysis [25, 26] to analyse pen portraits (narrative summaries of transcripts) [27] derived from semi-structured interviews with paramedics. Semi-structured interviews were used to enable in-depth exploration of the paramedics’ experiences and to provide a safe space for discussing sensitive and emotional topics [28].

### Sampling and recruitment

A purposive sampling approach was employed, aiming to capture a diverse range of OHCA events and patient profiles by recruiting paramedics within two Ambulance Services which were structured differently and which included both rural and urban environments. Our sample included paramedics with a range of exposure to cardiac arrest. Ambulance Services identified adult cardiac arrests that had occurred during the previous fortnight. Research Paramedics approached qualified, practising paramedics who had attended these cases, inviting them to participate in the study. The target number of participants was set at 30 by the research team and the wider study Patient and Public Involvement (PPI) panel with a view to continuing recruitment until the study team agreed that meaning saturation, understood as the point at which no new insights were being generated, had been reached [29]. The sampling method aimed to capture variations in paramedic practice and experience across the breadth of the Ambulance Services. Participants were offered a shopping voucher worth £40 in consideration of their time.

### Data collection

The project team developed an interview topic guide with input from MAS (a senior research paramedic). Initial questions were designed to encourage participants to describe the specific cardiac arrest event, with subsequent questions probing for explanations around decision-making and descriptions of participants’ expectations, dilemmas, challenges, values and beliefs, and learning points. Later questions asked for a discussion of participants’ general thoughts about OHCA.

All interviews were conducted remotely (via telephone or Microsoft Teams) by CH (a research psychologist), KE (a medical anthropologist) and JW (a health sciences researcher). Interviews took place between March and July 2022. Interviews were recorded on an encrypted digital recorder and professionally transcribed verbatim. Where appropriate, interviewers recorded reflections about the interview context and process, to enable more accurate analysis of data and provide learning points for future interviews. Initial interview transcripts were reviewed by the core study team and the interview guide refined. In addition to CH and KE, the core team included GG (a research psychologist), FG (a medical sociologist) and A-MS (a clinical ethicist).

### Data analysis

Transcripts were checked by GG (a research psychologist) for accuracy and anonymity, allowing familiarisation with interview data. GG created pen portraits from the checked transcripts, adopting the procedure recommended by Sheard & Marsh [27]. This method is effective for summarising interview data in cross-sectional studies [30], as it enables comparisons between participants whilst incorporating sufficient detail for themes to be developed. Between three and seven pages long, pen portraits were structured to capture background information, discussion of specific events, and general reflections. They were populated with content aiming to summarise all relevant text in a narrative format. The process was akin to a narrative form of detailed, inductive thematic coding [25]. The pen portraits incorporated direct quotes where these enhanced content through clarification or illustration. This approach facilitated subsequent higher-level coding of pen portraits relating to explicit and implicit factors moderating uncertainty and decision-making. Higher-level coding was also informed by uncertainty and decision-making literature. NVivo Pro 12 was used for higher-level coding. When ten pen portraits had been coded, codes were grouped into candidate themes. Following consultation with the research team, a revised thematic framework was developed from these candidate themes, around which subsequent deductive coding was based. Once coding was complete, the themes were discussed among the team and further refined [26].

### Rigour

Care was taken to recognise and minimise misrepresentation of interview data (e.g. through over-generalisation or over-representation of extreme quotes) using reflexive note-taking to identify and address bias whilst creating pen portraits. The structure and content of three pen portraits was approved by the core study team prior to continuing to create pen portraits. Pen portraits were re-checked for accuracy prior to coding in NVivo, referring back to original transcripts where this clarified content. Coding was regularly discussed at weekly meetings with the core study team and findings were discussed at monthly meetings with wider team members MAS (a senior research paramedic) and GDP (a critical care specialist). A subset of 25% of pen portraits was coded independently by two research team members, KE and CH, who had conducted interviews. Coding consistency was qualitatively assessed, with suggestions discussed and incorporated into the analysis. Candidate themes were discussed, modified and agreed with all research team members prior to coding the remaining data. Concurrent to this process, an ongoing record of researcher observations relating to uncertainty and decision-making enabled a more holistic understanding of the findings.

### Reflexivity

Reflexive processes were incorporated into data collection and analysis through recording and responding to reflections on interview procedure and data analysis; considering potential bias resulting from the emotive nature of the topic during regular research team meetings; and reflecting on stakeholders’ reactions to a presentation of initial findings at a stakeholder meeting comprising paramedics, emergency department staff, members of the public with experience of OHCA, and researchers.

### Ethics

This study was conducted in accordance with the principles of the UK Policy Framework for Health and Social Care Research. The East Midlands – Derby Research Ethics Committee (reference: 19/EM/0358) approved the study. Potential participants were sent a participant information sheet and consent form via email. All participants provided verbal informed consent, formally recorded by the researcher prior to the start of the interview. Participants have been pseudonymized and identifying details have been removed from this manuscript.

Research participants were talking about a sensitive topic but one that they deal with within their professional roles and for which they have professional support mechanisms. The interviewers were non-clinical researchers. They worked as a team providing peer support and met with FG and A-MS (both experienced clinicians and researchers) weekly during data collection to discuss interviews’ content and its impact on them.

## Results

### Participants and events

Thirty-one paramedics participated in interviews, seventeen from one region and fourteen from the other. Interviews lasted between 28 and 96 min (mean duration 62 min). Twenty-six participants had more than two years’ experience as qualified practising paramedics, with ten of these having over ten years’ paramedic experience. Based on their qualifications, we classified fifteen participants as ‘senior paramedics’ and refer to quoted paramedics as ‘paramedic’ or ‘senior paramedic’. Participants reported the frequency of their attendance at OHCA to range from one or two per year to several per week. Solo responders and senior paramedics reported attending more cardiac arrests than those on double ambulance crews.

Thirty-two OHCA events were described in detail (one paramedic described two separate events). Participants were leaders or co-leaders of the resuscitation in twenty-six cases. Twenty-five events were described by participants as being managed within standard guidelines. All but one of the seven events that were described as having been managed outside standard guidelines involved seeking senior support. The one exception involved a paramedic with sufficient seniority to manage the event autonomously.

Five resuscitation attempts occurred in a public location. Four of these resulted in hospital transfer and one was ceased at the scene. Twenty-seven OHCA events occurred in private residences, with resuscitation ceased at the scene in sixteen of these patients.

### Thematic findings

Four overarching themes were developed, describing paramedic uncertainty and identifying factors influencing paramedic assessments of survivability and decision-making in OHCA (Table 1). The first theme explores the uncertainties that paramedics described encountering within resuscitation guidelines during different stages of an OHCA event. The second theme investigates personal, practical and relational factors outside current guidelines that were perceived to influence decision-making during management of an OHCA. The third theme describes how the holistic assessment of a combination of factors typically informed assessments of survivability and aided decision-making. The fourth theme captures factors influencing OHCA management that operate over longer time periods and/or on broader scales than that of the individual event.

**Table 1:** Summary of themes relating to uncertainty and decision-making in OHCA

Theme	First-level sub-theme	Second-level sub-theme
1. Uncertainties encountered in resuscitation guidelines	a. Uncertainty in starting resuscitation.	
	b. Uncertainty around continuing resuscitation	
2. Personal, practical and relational influences on resuscitation decision-making	a. Personal factors: paramedic experience, beliefs and values.	i) Paramedics’ attitudes to patient age and quality of life. ii) Paramedic experience and seniority.
	b. Practical factors: environment, resources and logistics moderate decision-making.	i) Scene accessibility and mechanical compression influence transport decisions. ii) Patient-related factors can cause practical challenges. iii) Resources, geographical location and dispatch decisions.
	c. Relational factors: the influence of others on paramedics’ decisions.	i) Family and bystanders affect decision-making. ii) The influence of other Ambulance Service staff on the scene.
3. Holistic perspectives inform survivability assessments and aid decision-making.		
4. Factors that can moderate decision-making over a longer time period/wider scale than individual OHCA events.		

## Uncertainties encountered in resuscitation guidelines

All participants described resuscitation guidelines to be the main drivers of their decision-making. Guidelines are based on clinical criteria, time since arrest (downtime), bystander cardio-pulmonary resuscitation (CPR) and anticipatory treatment recommendations (e.g. Do Not Attempt Cardiopulmonary Resuscitation (DNACPR) forms, Recommended Summary Plan for Emergency Care and Treatment (ReSPECT) forms, and advance decisions). Participants felt that guidelines provided direction, protection, and confidence. All participants followed guidelines to the best of their ability, using senior support where necessary. The check-sheet approach was generally felt to be helpful, described as “clear cut and easy to follow” (Participant 4) and “a functional tool and a good aid” (Participant 11), particularly for paramedics not in senior roles. Nonetheless, common areas of uncertainty were identified in guidelines: “I think some of the [guidelines] occasionally leave grey areas… that leaves room in people’s minds for interpretation which can be a bit dangerous, depending on the person” (Participant 27, senior paramedic).

The degree of paramedic uncertainty evident in interviews varied according to differences in paramedic experience and the availability of remote senior support, with more senior paramedics tending to report greater confidence in their ability to interpret guidelines. Uncertainties in guidelines were encountered at all stages of OHCA management: when starting, making treatment or transport decisions, and ceasing resuscitation.

### Uncertainty in starting resuscitation

Upon arrival to an OHCA, paramedics frequently described the decision to commence resuscitation (or continue if bystanders had begun CPR) as challenging, with one participant perceiving decisions to “vary from clinician to clinician” (Participant 4). Uncertainties at this stage of resuscitation decision-making derived from difficulties in applying guideline criteria, often due to lack of information about a patient’s medical history or advance wishes. These uncertainties could have both clinical and ethical dimensions. For example, doubts often arose when paramedics considered that resuscitation would be unsuccessful but guideline criteria required them to start. This could occur if bystander CPR had begun but its effectiveness was unclear, or when a patient’s time of collapse was difficult to determine. The practical need for clarity was described by Participant 28, a senior paramedic: “Whether [the cardiac arrest] was witnessed or unwitnessed is a, is a big factor because you can really pinpoint a time then. The absence or presence of bystander CPR and was it effective.” Furthermore, interpreting definitions of criteria such as “end stages of a terminal condition” could be problematic, particularly when a patient’s medical history was unavailable, leading to calls for “a bit more clarity as to …what should be interpreted as an advanced stage of a terminal condition. Because age is ultimately terminal… frailty is ultimately terminal” (Participant 19, senior paramedic). This was highlighted by Participant 31, a senior paramedic, who described their uncertainty and discomfort in attempting to resuscitate a centenarian who had no advance directive:

I really didn’t know what to do, I really didn’t wanna start on her, you know, why would I want to resus a 101-year-old? So… I called up the advanced paramedic desk and said, “Look, this is the situation,” and they said, “I think you should start, just until we get more information on whatever or at least do the 20 minutes.” So we did, we started.

The patient did not survive, and the participant felt that the experience was “not a very dignified way to go” for the patient.

The presence of a document specifying a recommendation that CPR should not be performed – such as a ReSPECT or DNACPR form - usually clarified uncertainties around starting resuscitation. However, these documents could compound uncertainty when forms were unclear, undated, or incomplete: “Especially since the beginning of the pandemic… they dished out ReSPECT forms like penny sweets. Unfortunately. …a lot of them ReSPECT forms haven’t been reviewed… or haven’t got review dates on them” (Participant 22, senior paramedic).

### Uncertainty around continuing resuscitation

This was particularly evident in OHCA patients whose hearts showed a PEA rhythm. Paramedics frequently described being unsure whether and when patients in PEA rhythms should be transported to hospital or treated at the scene; whether, when and how the guidelines allowed them to cease resuscitation; which paramedics had autonomy to do so; and whether guidelines had changed. Those who were uncertain always erred on the side of caution, either transporting patients with ongoing CPR or seeking senior support for their decisions at the scene. Variations in which of these options was taken were apparent according to the level of senior support available and the experience of the paramedic. The uncertainty accompanying PEA decision-making was often experienced as challenging. For example, Participant 25 recalled the dilemma they faced when another crew questioned their decision to convey a patient in PEA to hospital. The other crew asked:

Oh, do you think we should run with this? Because actually she’s in PEA but is really wide complex… We know that it’s not really compatible with life, and we know they’re just going to call it at the hospital. Should we just call it now?

The participant continued to transport the patient because they “just felt a little bit uneasy calling it on the scene.” Later in the interview they reflected “I think we should be able to call it but I think there needs to be a… framework around it,” acknowledging the complexity of the situation and the dangers of using PEA alone to indicate survivability. This reflects frequent calls from participants for clarity on PEA decisions, with a wide range of opinion on whether paramedics should have the autonomy to decide to stop CPR in PEA cases.

Further uncertainties in ‘transport or treat’ decisions commonly arose from complexities associated with patients’ unstable responses to adrenaline. This was particularly challenging when paramedics were assessing the risks of a patient re-arresting during extrication and transport, as experienced by Participant 10, who sought senior advice for a patient:

[She] was getting so many ROSCs, despite how long it was lived, [senior support] weren’t particularly happy with deeming anything to be end-of-life… They said… “start looking at finding some extrication. Then take the patient to hospital.” Problem was, every time this happened, she rearrested.

Scepticism about patient survivability often accompanied such cases:

If we do get them out of asystole it’s because of the adrenaline that we get them out of it… and then they become adrenaline dependent and then when we hit our max dose of adrenaline we can’t do anything more for them and then [the patients] die” (Participant 31, senior paramedic).

## Personal, practical and relational influences on resuscitation decision-making

When faced with uncertainty in their interpretation of resuscitation guidelines, paramedics described a range of non-clinical factors that could affect their decisions. These are explored in the following sub-themes.

### Personal factors: paramedic values and experience

#### Paramedics’ attitudes to the complexity of age, frailty and quality of life

Despite the absence of age within current guidelines, the majority of paramedics said that patient age affected their assessments of survivability, although participants’ definitions of “old” and “young” tended to be ambiguous. Older patients often prompted searches for anticipatory treatment recommendations such as ReSPECT or DNACPR forms: “When I see how old they are I’m, I’m always thinking have they got a ReSPECT form” (Participant 9).

The influence of patient age on paramedic decision-making was evidenced both through explicit statements and implicit accounts. Younger patients inspired a sense of urgency and were more likely to be transported to hospital, worked on for longer, and require senior support. Participant 26 recalled their reaction upon disembarking from the ambulance and discovering a patient was younger than expected: “And [a bystander] said, “Oh, she’s in her fifties.” And I, both my crew mate and I looked at, looked at each other and were like, “Oh, okay.” So we kind of like hurried a bit quicker.” Age-related responses were more consciously acknowledged by Participant 6, who commented that “paramedics get a little more apprehensive calling arrests [deciding to stop resuscitation] the younger they get”. However, overt descriptions of age influencing survivability assessments were often accompanied by a degree of discomfort, with acknowledgement that health status varies within age bands and that exceptions do exist: “He made a full recovery apparently which is very unheard of, considering how elderly he was. Like I said before, how do you draw the line as to who’s gonna recover and who isn’t?” (Participant 30, senior paramedic).

Many paramedics reasoned that old age was associated with low physiological reserve, co-morbidities and reduced survival or recovery chances. Participant 19 (a senior paramedic) said:

I’m not gonna put an age on it. But there comes an age where, even if your heart maybe somehow survives the insult, you’re never gonna be discharged from hospital as the same person that you went in as. And - do people want that? That’s the big bit of work that needs to happen, is, what do people want?

Participants’ personal beliefs about age often linked with assumed quality of life, dignity, best interest, or years left to live for patients. Reflecting on the decision to continue or terminate resuscitation, Participant 10 felt “the age ends up being such a determining factor, not just about whether we should continue but also someone’s ability to bounce back and the quality of life they’d have afterwards.” Another participant suggested that paramedics may identify more with younger patients because they were of a similar age, thereby influencing their decisions to convey patients to hospital:

You could have a 30-year-old with, like, comorbidities but they’re still only 30. And I think age puts a little bit of a factor into it, whether it’s ‘cause they’re a similar age to the paramedics and it’s a bit like a, a social, a social thing but I think they’re the ones that tend to maybe go to hospital (Participant 6).

Many participants talked about being wary of allowing subjectivity to influence decisions, described by Participant 14 (a senior paramedic) as “a bit of a moral dilemma… yeah, thoughts and beliefs I think, it’s, it’s a bit of a tricky one… it becomes a bit dangerous really when we start to kind of put our own spin on things.”

#### Paramedic experience and seniority

Uncertainty and lack of confidence from less experienced paramedics often resulted in more senior or experienced paramedics making resuscitation decisions. This was illustrated in one participant’s account of reassuring a less experienced crewmate:

[They hadn’t] been to a cardiac arrest for a long time… so [they were] a bit under-confident going into that job and was kind of looking to me… When [they were] placing the airway [they] just wanted me to check, “Is this right? Do you think, are you happy with this? Are we okay?” (Participant 25).

Some participants thought guidelines were particularly useful to address uncertainty for inexperienced paramedics. Participant 28 (a senior paramedic) described guidelines as “especially for junior members if they don’t have a clinical team leader,” contrasting this with senior colleagues who had “more freedom to kind of use your experience of previous cardiac arrests, the quick access to an on-call system, ultrasound and that feeds more into the decision.”

Factors such as fear of reprisal, and feeling stressed or tired, were mentioned as an issue when paramedics faced uncertain situations. For example, Participant 10 described how fatigue following a seventeen-hour shift when attending a complex event resulted in them “questioning things you never normally questioned” and seeking senior support for their decisions.

Although experienced paramedics tended to show more confidence in making termination decisions, previous experience of exceptional OHCA events could instil a degree of caution. This was illustrated by Participant 1, a senior paramedic whose experience of a “one in a zillion” case of spontaneous recovery following cessation of prolonged resuscitation led them to comment that “it makes you think long and hard about that decision, and it makes you make sure you are making a safe a decision as you can.”

### Practical factors: environment, resources and logistics moderate decision-making

#### Scene accessibility and mechanical compression

For patients whose condition might deteriorate during extrication and transport, access options were particularly influential in decision-making. Some events involved patients who had arrested in hard-to-reach places, such as cluttered houses or up flights of narrow stairs. In such situations, the availability of a mechanical compression device could enable effective compressions to continue during conveyance, reducing risks to patients and clinicians and influencing transport decisions:

An automatic chest compression machine… means that you can move people whilst that is on, and it means they will still be getting chest compressions completed, whereas generally, if we’re going downstairs, we can’t do that so then you think, well, there’s no point moving them because for that whole period of going down the stairs, all the work that you’ve done for the last however long is wasted. (Participant 30, senior paramedic).

#### Patient-related factors

Dilemmas could arise from patient-related factors such as large patient size, bodily discharge or clothing that was difficult to remove. This added a layer of complexity which could cause delay and uncertainty in transport or treatment decisions. For example, the risks to patient and paramedics of transporting heavier patients increased the complexity of extrication decisions. This was experienced by Participant 14 (a senior paramedic), who decided to seek senior advice when unsure whether to convey a patient in cardiac arrest to hospital:

I spoke to the… advanced paramedic on the radio […] he was in asystole and the history was quite, because it was quite unclear I wasn’t really sure if they, going to hospital was necessarily the right thing to do in this situation and the other factors were that he was up six flights of stairs and he was a, he was a large gentleman. I knew it was going to be a really, really difficult removal […] we couldn’t get him down the stairs because he was too heavy, so we had to wait for the fire brigade.

#### Resources, geographical location and dispatch decisions

Treatment and transport decisions were influenced by other logistical moderators including staff resources, medical equipment, pandemic-related delays, journey times and hospital facilities. Participant 19 (a senior paramedic) described how logistics intersected with arrest aetiology in potentially reversible cases, such that journey times and hospital facilities influenced transport decisions: “[For] cardiac arrest[s] because of a, an MI [myocardial infarction, a reversible cause of arrest], and you’re close to a hospital that can resolve an MI, then you would very quickly look at moving them to that hospital.” These factors were thought to be less relevant to decisions involving patients with irreversible conditions for whom “there isn’t anything else that hospital’s gonna add.”

The accuracy of information from initial emergency calls, and subsequent ambulance dispatch decisions, could affect participants’ preparation and decision-making upon arrival at an OHCA event. Participant 15 recalled a mis-categorised event for which, instead of the usual double crew, they were dispatched as a solo responder, necessitating the decision to recruit a bystander to help initiate basic life support whilst waiting for backup. They recalled the challenge of trying to “do everything with lots of hands you don’t have.”

### Relational factors: the influence of others on paramedic decisions

#### Family and bystanders

Relatives, friends and bystanders could influence paramedic decisions by providing assistance and valuable information; as sources of emotional pressure; and as recipients of support both during and after their traumatic experience. The importance of family and bystander considerations was emphasised by the majority of participants. Participant 24 (a senior paramedic) summarised this by reflecting that “we should be making these decisions based on the clinical picture and potential outcome; however, there are social and humanitarian reasons that you may move somebody to hospital.” Bystander pressure could both instigate decisions to convey patients to hospital, and delay decisions to terminate resuscitation at the scene. Participant 29 reflected:

Considering the family members’ emotions is probably the hardest factor as to stopping … I’d maybe do it longer, a little bit longer once we’d made the decision, just so that the family members can potentially come and be in the room when we cease resuscitation.

#### Other Ambulance Service staff on the scene

Other staff played a key role in decision-making and were highly valued by participants. Accessing senior support, sharing decisions and consulting team members helped participants by providing direction, checking nothing had been missed, and increasing confidence in the decision-making process. Staff were generally in agreement with one another, although occasional conflicts of opinion were reported to increase uncertainty. Considerations of staff welfare, safety and legal protection could also affect participants’ decision-making. A senior paramedic summarised the interplay of staff attending an event as follows:

The crews do all of the hard work and then as the advanced practitioner you arrive, can take a complete overview, gain a bit more history, look at the bigger picture and then with the team add your information into their information and make a decision together. I think it’s so crucial especially in the advanced ROLE to share decision making because otherwise as a solo responder, that’s quite heavy (Participant 24, senior paramedic).

Team familiarity, along with effective leadership and role allocation, were described to inspire trust and improve co-ordination of decisions, as highlighted by Participant 23:

We’ve worked together for the last nearly four years, we just know how each other work… And it does work a lot better… And obviously when you get an influx of new staff you’ve got to build up, you know, build that trust up over time.

## Holistic perspectives inform survivability assessments and aid decision-making

OHCAs were described as unique, complex and context-dependent. Although individual factors influenced paramedic decisions, management of resuscitation was generally perceived to be an integrated process:

It’s all, all these factors that actually aid your decision making, it’s not just about the ReSPECT form, comorbidities, it’s about how long was it before that person called us? Was CPR started? And then it’s took us how long to get there? And yeah, it’s a lot of decision making. (Participant 23)

Holistic decision-making was sometimes implicitly evident in participant accounts, described in terms of combinations of factors moderating decisions, particularly during complex events. Other participants explicitly recognised the holistic nature of resuscitation decisions or used concepts such as best interests, risk-benefit, survivability and futility to portray the multiple influences on decision-making. In all accounts, guidelines were perceived to intersect with non-clinical moderators of decision-making. Participant 19 (a senior paramedic) described the challenges that this involved:

Statistically we know the answer. And then holistically, actually we see both sides of it. We see people who are resuscitated who, no one wants them to be resuscitated. And we enforce a, “We’re not resuscitating this person,” decision on people who do want stuff. But clinically they can’t, they’re not gonna survive. So it’s incredibly difficult.

When considering how non-clinical factors could be incorporated into guidelines, Participant 1 (a senior paramedic) acknowledged that “the bit that is difficult to protocolise is the kind of experience bit of it, and the bringing other factors including family decisions, patient decisions… all of that you have to amalgamate.” Sharing decisions with other clinicians was seen as a potential solution: “As we go forward, actually, it’s recognising that sometimes that decision is best as a supported decision.”

## Factors that can moderate decision-making over longer time periods/wider scales than individual OHCA events

Participants identified a number of factors which could moderate decision-making over a longer time period or at a wider scale than that of the individual OHCA event. These were described as influencing future OHCA management via feedback from stakeholders including health services personnel, patients and bystanders at previous events, and the public.

Post-event debriefs and interactions with hospital staff during patient handovers were perceived to influence paramedics’ attitudes to future OHCAs by providing learning points and affecting morale. Participant 27 (a senior paramedic) emphasised the importance of regular reflection in practice by suggesting “always have a debrief… in an absolutely judgement free zone” because if there is “a tiny little lesson that can be learned … that makes them 2% more efficient, that 2% could be the difference later on in their career.”

On a wider scale, participants thought regular training, an evolving service, dissemination of research, and systemic changes - particularly with regards to communication between paramedics on scene, remote support and hospitals - affected resuscitation protocol and practice. These factors were often manifested in interviews as problems or suggestions for improvement that could affect decision-making. For example, Participant 1 (a senior paramedic) felt that investment in effective remote support systems allowed paramedics to “make solid evidence-based decisions” in nuanced cases; and Paramedic 28 (a senior paramedic) suggested that the development of more defined pathways between paramedics and hospital life support services would give paramedics confidence in making conveyance decisions, commenting that “unfortunately, in my experience these pathways are not there yet, so it may even be a case of that I don’t make that phone call [to hospital] if I know it’s not going to happen.” Variation in treatment decisions from crews in adjacent regions when working together was mentioned as a problem by Participant 13 (a senior paramedic), describing OHCA treatment as “not very uniform sometimes” and suggesting “a good rollout of training and a very clear Resus[citation] Council guideline” should be followed by all Ambulance Trusts.

Other indirect ways of reducing paramedic uncertainty during OHCA management included raising public awareness of OHCA survival rates and improving public engagement with advance decision-making, to enable clarification of patients’ wishes for or against resuscitation. When considering the value of encouraging ReSPECT forms for older patients with multiple care needs, Participant 7 commented that “maybe when you get to a certain age, a doctor should be talking to you about ReSPECT”. Participant 31 (a senior paramedic) thought a solution to unnecessary and undignified resuscitation attempts to be “a lot more public education” around advance decision-making “because members of the public have absolutely no idea” about the legal aspects of resuscitation.

Increased public ability and willingness to perform bystander CPR was also believed to influence paramedics’ confidence when making decisions to commence or continue resuscitation upon arrival at an event. The importance of bystander CPR in affecting decision-making was illustrated by Participant 4, who recalled that a “tricky decision” to start resuscitation was clarified largely “because the patient had effective [bystander] CPR in place” such that “there wasn’t any signs of rigor.” However, Paramedic 25 felt this was not a common occurrence: “It seems as though not many people actually start CPR before we arrive. People are too scared to do it wrong,” with Paramedic 18 (a senior paramedic) emphasising that “education’s so important and trying to promote CPR and public access defib [defibrillators].”

## Discussion

In this interview-based study with paramedics who have led resuscitation decision-making in OHCA events we found that, whilst guidelines directed decision-making, areas of uncertainty remained. These related primarily to criteria for starting resuscitation; managing patients in a PEA rhythm; and managing unstable responses to adrenaline. Non-clinical personal, practical and relational factors were found to moderate paramedics’ assessments of survivability and decision-making. Paramedics’ attitudes to the complex interactions between patient age, frailty, quality of life and survivability were a notable influence. Non-clinical moderators could alleviate uncertainty when aligned with guidelines or propagate uncertainty when in conflict. Propagation of uncertainty did not always hinder decisions and could sometimes inform them, with participants taking a cautionary approach and seeking support when uncertain. Paramedics commonly described taking a holistic perspective, integrating guidelines with non-clinical moderators of decision-making. Furthermore, they identified indirect influences on longer-term OHCA management such as public awareness and systemic efficiency, reflecting findings from other studies [31, 32].

Uncertainties experienced in OHCA management arose from a number of sources: *probability* (indeterminacy of future outcome), *ambiguity* (imprecise, conflicting or inadequate information) and *complexity* (multiplicity of causal factors and interpretive cues) [13]. Assessing the probable outcome of a resuscitation could be an uncertain process and there was often incomplete or ambiguous information about a patient such as unknown downtime, unclear patient wishes and inadequate medical history. Uncertainty arising from complexity could be experienced in a clinical capacity such as repeated rhythm changes caused by unstable responses to adrenaline, which could be confusing to manage; and could be compounded by non-clinical factors such as difficult access increasing the risk of compromising resuscitation during extrication. Paramedic uncertainty could be clinical and practical in nature, but at other times it took on an ethical dimension. This was particularly evident when personal values were at variance with guidelines. While research on ethical considerations in resuscitation decisions is sparse to date, recent work in Denmark by Milling et al. [11] suggests that paramedics’ ethical considerations can play a substantial role in OHCA management. Furthermore, paramedics’ sense of personal connection with patients and families has been found to impact the coping strategies used in critical situations [33].

Participants’ descriptions of uncertainty in guideline criteria reflect findings from previous studies [5, 10, 34]. Moreover, previous work has identified similar non-clinical factors moderating paramedics’ uncertainty and decision-making - including attitudes to patient age – and found that their complex interaction can lead to conflicting priorities and variation in practice [4, 5, 8, 9]. The PreDem model of resuscitation decision-making envisages factors influencing paramedic decision-making as facilitators or challenges [7]. Similarly, this study finds that interactions between clinical and non-clinical factors in OHCA can both challenge and facilitate decision-making and that the complexity of their interactions leads to unpredictable, non-uniform responses.

Our findings of the strategies paramedics described adopting to mitigate uncertainty resonate with findings of naturalistic decision-making studies that used the RAWFS heuristic [18–21]. For example, reducing uncertainty through seeking information on patients’ medical status and wishes was commonly described as a coping strategy during OHCA management, with participants using assumption-based reasoning to adopt a precautionary approach to treatment when this information was unavailable. Balancing the pros and cons was also frequently described as a process used to manage uncertainty in transport-related decisions involving assessing relative risks to patients, relatives, paramedics and wider society.

Similarities identified between our findings and those of these studies suggest that future research could focus on using the RAWFS framework to develop a detailed examination of how these processes are applied during resuscitation decision-making, and thereby contribute to the development of a robust evidence base to inform resuscitation policy training.

### Strengths and limitations

A strength of the study was its inclusion of paramedics from two Ambulance Trusts covering diverse populations, and paramedics with varying levels of experience. The interviews explored recent cardiac arrest events attended by the participants who grounded their responses in recent reality. The study was limited by its focus on paramedics who were designated as the main decision-makers in the cardiac arrest event so the views of less experienced paramedics were largely unexplored; it is therefore possible that our findings may be more relevant to paramedics who frequently attend OHCA. This focus led to the recruitment of many paramedics who attended several cardiac arrest events each week, meaning that, in six cases, participants were unable to recall the event for which they had been recruited. These participants chose another recent event to discuss but it is possible the event selected was more memorable and hence less representative.

## Conclusion

NHS paramedics attending OHCA events experienced areas of uncertainty at all stages of the resuscitation process. These arose from indeterminate, ambiguous and complex sources; had clinical, practical and ethical dimensions; and were experienced differently based on paramedic seniority and resource availability. Paramedics perceived uncertainty as moderated by a variety of clinical and non-clinical factors that were integrated into a holistic decision-making process, incorporating coping strategies resonant with those described by the RAWFS heuristic [17].

The identification of uncertainties in paramedics’ interpretations of resuscitation guidelines, together with their descriptions of both clinical and non-clinical factors moderating OHCA management, implies variation in paramedic practice within the UK. Paramedics’ accounts of how they assess survivability and make resuscitation decisions highlight the importance of acknowledging and addressing uncertainties in resuscitation guidelines, and of further exploring the complexity of interactions between clinical and non-clinical factors moderating paramedic responses to OHCA management, with a view to increasing consistency and optimising patient care.

## Data Availability

Although data in this qualitative study were pseudonymised, it is possible that with access to raw data individuals might be identifiable from participants’ descriptions of events. The data are highly sensitive and not suitable for sharing beyond what is contained within the manuscript. Further information regarding this can be obtained from the corresponding author.

## Declarations

### Disclaimer

The views expressed in this publication are those of the authors and not necessarily those of the NIHR or the Department of Health and Social Care.

## Abbreviations

OHCA: Out-of-Hospital Cardiac Arrest
UK: United Kingdom
ROLE: Recognition of Life Extinct
PEA: Pulseless Electrical Activity
RAWFS heuristic: Reduction of uncertainty, Assumption-based reasoning, Weighing pros/cons of competing alternatives, Forestalling/hedging, Suppression (17)
ReSPECT: Recommended Summary Plan for Emergency Care and Treatment
DNACPR: Do Not Attempt Cardiopulmonary Resuscitation
CPR: Cardiopulmonary Resuscitation
ROSC: Return of Spontaneous Circulation
